# Study protocol for a feasibility study of an online educational programme for people working and living with persistent low back pain

**DOI:** 10.1186/s40814-023-01382-3

**Published:** 2023-09-04

**Authors:** Joanne Marley, Caroline Larsson, Flavia Piccinini, Sarah Howes, Elisa Casoni, Eva Ekvall Hansson, Suzanne McDonough

**Affiliations:** 1 Faculty of Life and Health Sciences, School of Health Sciences, Ulster University, Northland Road, Londonderry, Northern Ireland; 2 Division of Physiotherapy, Department of Health Sciences, Lund University, 22240 Lund, Sweden; 3 Centre for Socio-Economic Research On Ageing, IRCCS INRCA-National Institute of Health and Science On Ageing, 60124 Ancona, Italy; 4 Clinical Unit of Physical Rehabilitation, IRCCS INRCA-National Institute of Health and Science On Ageing, 60127 Ancona, Italy; 5 School of Physiotherapy, Royal College of Surgeons in Ireland, Dublin, Ireland

**Keywords:** Back pain, Web intervention, Self-management, Digital education programme, MOOC

## Abstract

**Background:**

Low back pain (LBP) is the main cause of activity limitation and work absence across the world, leading to a high social and economic burden for individuals, families, the labour market and society. The overall aim of this multicentre study is to test *the usability*, *acceptability* and *feasibility* of an evidence-based, digital education programme for people living and working with persistent LBP who are in sedentary or physically demanding jobs and need advice on ergonomics, self-management of pain and healthy behavioural strategies.

**Methods:**

This is the protocol of a multinational, multicentre, prospective uncontrolled feasibility study targeting people with persistent LBP in Lithuania, Northern Ireland, Italy, Sweden and Portugal. Eligible participants will be offered the opportunity to use the MyRelief educational platform as part of their care and will undergo evaluations at baseline (enrollment) and 1-month follow-up. Feasibility will be assessed using measures of recruitment and retention, intervention engagement, outcome measure completion rates and within-group effect sizes in response to the digital education programme.

**Discussion:**

This study will identify the challenges and implications of delivering a digital training programme in advance of potentially delivering the programme via an online educational platform available on mobile devices. The findings will inform the design of a future randomised controlled trial if it proves feasible.

**Trial registration:**

ClinicalTrials.gov, NCT04673773. Registered 17 December 2020.

**Supplementary Information:**

The online version contains supplementary material available at 10.1186/s40814-023-01382-3.

## Background

Low back pain (LBP) is the main cause of activity limitation and work absence across the world, leading to a high social and economic burden for individuals, families, the labour market and society [1, 2]. This is particularly true for adults, employed in sedentary or arduous/strenuous jobs, whose work conditions may negatively influence this health problem and lead to severe persistent LBP [3]. Pain management is one of the most neglected aspects of healthcare, and people living with persistent LBP are often left without specific information, guidance and care from healthcare systems [4]. Alternative treatments for managing or decreasing pain include physical therapy and exercises, but awareness and adherence are quite low [5].

Self-management is the key recommendation for managing LBP and is found to have a significant effect on pain intensity and disability in people with persistent LBP [6]. In a systematic review from 2017, web-based interventions were shown to hold the potential in supporting self-management strategies in LBP [7], although the heterogeneity among studies made it difficult to draw any conclusions regarding effectiveness. An ongoing study (SupportBack2) investigates an Internet intervention designed to support patients to self-manage their LBP following consultation in primary care [8]. While previous studies are targeting patients in care, massive open online courses (MOOCs) can be used targeting a wider audience. MOOCs are increasingly being offered in the area of health and medicine education [9] and can when offered through reputable institutions provide valuable access to reliable information without the constraints of time, geographical location, or level of education [10]. MOOCs also have the potential to increase the health literacy of the public with regard to the prevention and treatment of chronic conditions [10, 11], but little is known of its potential to support self-management strategies in LBP. Therefore, this novel study will investigate the delivery of a MOOC in a multicultural context.

This paper describes the research protocol of an uncontrolled pilot study testing the feasibility of a multi-language digital education programme called MyRelief to inform a future randomised controlled trial that would be delivered via a MOOC.

## Aims and objectives

### Aim

This study aims to explore the usability and acceptability of the digital intervention and the trial feasibility of a digital education programme for people with persistent LBP.

### Objectives

(i) The usability of the intervention will be measured with an industry standard tool to understand people’s ease of interaction with the digital education programme, and acceptability of the content and format of the digital education programme will be measured via qualitative feedback from users.
(ii) Feasibility of a future trial will be informed by the data on likely recruitment and follow-up rates in each country for a main trial, floor and ceiling effects and completion rates on outcome measures and likely within-group effect sizes in response to the digital education programme.
(iii) Compare uptake of, and engagement with the intervention, when delivered via an online educational provider of massive online open courses freely available to non-study participants

## Methods

**Fig. 1:**
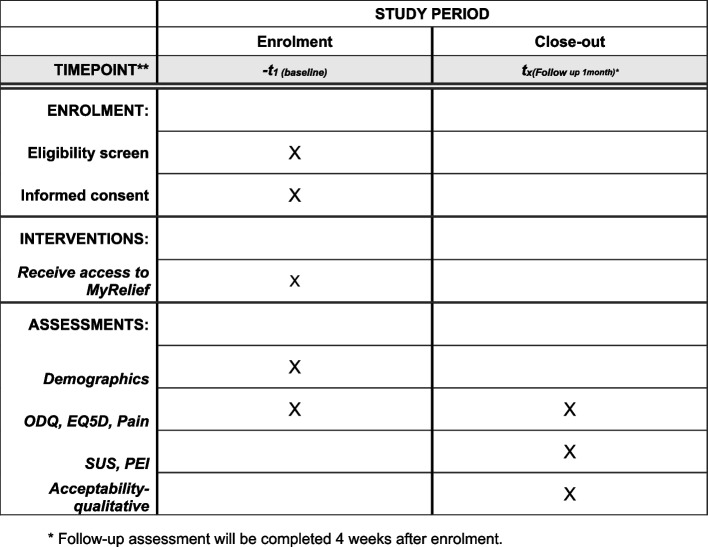
SPIRIT schedule of enrolment, intervention and assessment

This protocol was prepared in accordance with the SPIRIT 2013 guidelines for reporting protocols of clinical trials. A SPIRIT schedule of enrolment, intervention and assessment is outlined in Fig. 1, and a SPIRIT checklist has been included as Additional file 1.

### Study design

This paper is the study protocol of a multicentre, multinational feasibility study of a digital intervention called ‘MyRelief’, targeting people with persistent LBP in Lithuania (LT), Northern Ireland (NI), Italy (IT), Sweden (SV) and Portugal (PT). This prospective uncontrolled, intervention study will test the usability, acceptability and trial feasibility of a complex intervention, as defined by the Medical Research Council [12], to inform the design of a future randomised controlled trial.

Eligible participants will be offered the opportunity to use the MyRelief educational platform as part of their care and will undergo evaluations at baseline (enrollment) and 1-month follow-up. The study design complies with the Declaration of Helsinki and Good Clinical Practice guidelines. Ethical approval has been obtained in each country. Each partner will be responsible for the translation of materials into their own language.

This will be an uncontrolled trial without a second ‘standard care’ arm (which may be no care depending on the country) at this stage. We consider it more important to learn about the usability and acceptability of MyRelief, rather than differences between arms, and acceptability to randomisation.

### Participating centres

- University of Ulster, Ulster, NI
- Lund University, Lund, SV
- National Institute of Health and Science on Aging (IRCSS INRCA), IT
- Virtual Campus, Porto, PT
- Kaunas University of Technology, Kaunas, LT

### Study population

Participants will be adults with persistent LBP who register directly on the MyRelief study website. After reading information about the trial, participants will be asked to confirm, by clicking and checking boxes, that they meet the following eligibility criteria:

### Inclusion and exclusion criteria

#### Inclusion criteria

To be eligible for participation in the study, participants must be as follows:

1. Understand Lithuanian, English, Italian, Swedish or Portuguese, as relevant for each recruiting country
2. Be willing and able to give informed consent for participation in the study via the study website
3. Have persistent LBP, defined as pain between scapulae and gluteal region, with or without radiation towards one or both legs, present for at least 3 months
4. Be in employment

#### Exclusion criteria

Individuals may not enter the study if any of the following apply:

1. People with unexplained symptoms
2. People who have not seen a healthcare professional and received a diagnosis of persistent LBP
3. People who have been given a diagnosis of specific LBP
4. People with evidence of serious underlying pathology, such as a current diagnosis of cancer
5. Anyone who has not used or has no interest in using a computer, laptop, tablet or mobile phone

### Recruitment strategies

Recruitment of adult workers with LBP will begin in August/September 2020 with information/advertising on social media. Recruitment will also commence at each site when local ethical approval is obtained: from April 2021 (Northern Ireland), September 2020 (Sweden) and June 2021 (Italy). Recruitment will take place via several methods depending on each countries ethical approval, e.g. via health service waiting lists (Italy), email circulated to all staff and students at university (Sweden, Northern Ireland, Lithuania) or patients at a pain clinic (Portugal) or social media (Italy, Sweden, Northern Ireland, Portugal). For the latter approach, a Facebook page has been created with information about the project. Links to this page will be posted on Instagram, Twitter and other relevant forums.

Regardless of the method of recruitment, participants will access a link to the trial webpages where they will find the participant information sheet (PIS) and will be asked to complete online screening questions based on the inclusion/exclusion criteria and to complete an online consent form. Only participants fulfilling the inclusions criteria will get access to the online education. After completing the consent form correctly, they will be given access to the MyRelief which will be hosted on a study website. Participants will be allocated an identifier number via the website, and this will be linked to their eligibility criteria, consent form and ongoing use of the MyRelief. At registration, participants will also be asked to enter their email address and/phone number to enable reminders and contact/invitations for participating in the qualitative interview. Participants discontinuing the programme will be contacted by email for follow-up data and invited to share their reasons for discontinuation. All data collected will be encrypted and saved in a secure online repository held by Lund University, with participant data only accessible by the local research team at each site.

#### Intervention

MyRelief is an online educational package that is available on mobile devices, such as a mobile phone or a tablet. It is built on best practices in e-learning, e-health and multimedia-based learning. The content of MyRelief has been developed through a synopsis of information gathered through focus groups with adult workers with LBP, conducted in each country. In addition, an evidence synthesis was generated by reviewing the most up-to-date clinical practice guidelines [13–16] for LBP. The training is designed in short modules (10–12 min per module) that would be possible to complete during a break in work. Engagement with the MyRelief is optimised by its design which uses short videos of workers with LBP and professional experts, as well as knowledge tests and short factual texts. Each research team was responsible for preparing the national language version of the training course, according to local customs and cultural sensitivity. In addition, each team included experienced LBP health professionals who supervised the fine-tuning of the content, avoiding negatively connoted terms when describing the condition. Given the funder’s requirements, MyRelief will also be available at the same time to non-trial participants hosted via a MOOC. This will allow us to make comparisons between participants uptake of, and engagement with MyRelief as part of a research study, or in a more open way via the MOOC.

##### Active ingredients of the intervention

MyRelief contains eight units which cover the following topics: Understanding LBP, physical activity and exercise in relation to LBP, psychological factors, sleep/nutrition, management of LBP at the workplace, communication with health care and other issues related to LBP. Each unit will include short factual texts, short videos (2–5 min) with LBP and professional experts, and knowledge tests. Each unit will take approximately 20 min to complete, so that the complete educational programme will take 2–3 h to complete. It will be possible to complete MyRelief at one time; however, users will be recommended to complete two units per week over a month. This will give people time to reflect on the information and how to apply it to their working environment and daily life. MyRelief will be hosted on a study website for trial participants in the five countries. Worldwide access for nontrial participants will be available via Udemy, a provider of MOOC, and will be free of charge.

#### Study outcomes

##### Trial feasibility

Feasibility will be informed by the data on likely recruitment and follow-up rates in each country for a main trial, floor and ceiling effects and completion rates on outcome measures, and likely within-group effect sizes in response to the MyRelief. Measurements will be made of (1) whether it was possible to recruit sufficient participants into the study within a particular time frame, (2) the number of MyRelief units completed by people who are recruited into the study(3), whether they are willing to complete outcome measures at baseline and the end of the intervention, and (4) usability scores of the system. Details of who takes part in the research and the rates of drop-out from the study will be recorded.

##### Clinical markers measured at baseline

1. Functional disability will be measured by the *Oswestry Disability Questionnaire (ODQ) version 2.1a (approval to use the questionnaire received from Mapi Research Trust)*. This has been shown to be a valid and reliable measure of pain and physical function in people with LBP [17]. The ODQ consists of 10 sections, each with six levels (with a maximum score in each section of five points) that assess the individual’s limitations in various activities of daily living. The sum of all 10 sections is divided by the total possible score and the result multiplied by 100 to generate a percentage score. Values range from 0 (best health state) to 100 (worst health state) with an average score of 43% identified for chronic back pain participants [17–19]. A minimum important change of between 10 and 12 points over time, or an improvement from baseline of between 20 and 30% for an individual, has been recommended [20].
2. Quality of life will be measured by *EQ-5D-3 l*, a self-administered questionnaire that assesses the participant’s health-related quality of life using a core set of five health-related quality-of-life items [21] (approved licensed online version). Health states are transformed to a single index using a scoring algorithm (TTO) derived from valuation tasks undertaken with general population samples. As algorithms are still not available for all involved countries, the UK value set will be used. The validity and reliability of *EQ-5D-3 l* are supported, and it has been recommended for use in LBP research [22].

##### Follow-up assessment

The follow-up assessment of the clinical markers will be taken again via the study webpage at the end of the intervention (1 month). At follow-up, all measures will be repeated (apart from sociodemographic information). We will attempt to follow all participants, including those who discontinue the intervention early. Also, at follow-up, an adapted version of the *Patient Enablement Instrument (PEI)* will be administered. The PEI is a six-item questionnaire designed to measure the individual’s ability to understand and cope with illness and life following a consultation with a general practitioner [23]. The PEI is considered ‘the gold standard’ for measuring enablement [24]. Given this, an adapted version of the PEI will be used, where the words ‘after this appointment’ and ‘as a result of your visit to the doctor today’ will be replaced by ‘after using the MOOC’ and ‘as a result of using the educational programme’.

##### Usability of the intervention at follow-up

The usability of the MyRelief intervention administered via the MOOC will be measured with an industry standard tool, *System Usability Scale SUS*, to understand peoples ease of interaction. The SUS is a simple, 10-item scale giving a global view of subjective assessments of usability. The selected statements in the SUS are measured on a 5-point Likert scale and cover a variety of aspects of system usability, such as the need for support, training, and complexity, and thus have a high level of validity for measuring usability of a system [25].

##### Acceptability of the intervention at follow-up

Users’ satisfaction with the system will be evaluated via qualitative semi-structured interviews and/or focus groups with a purposely selected sample of participants (4–6 individuals/country). ‘An interview guide have been developed and translated by each partner into their native language’. ‘An interview guide have been developed in English and translated by each partner into their native language’. All interviews and/or focus groups will be audio-recorded and transcribed verbatim for further analysis. Interpretation, synthesis and data reduction will be undertaken, applying an inductive content analysis approach. After familiarisation with the data, a coding frame will be developed to facilitate coding of key concepts related to acceptability of the platform, followed by identification of the relevant themes as they emerge.

#### Statistical considerations

##### Estimation of sample size

No formal sample size has been conducted in this feasibility study. Twenty-four to 50 participants have been recommended in order to estimate a standard deviation for an outcome measure [26, 27], so we will attempt to recruit up to 50 participants overall across all countries.

The proposed sample size should provide sufficient information on the usability of the online platform, given that research on the number of participants required for usability testing indicates that 5–10 participants are sufficient [28, 29] with some suggestion that multiple small tests are more valuable in allowing iterative changes to be made based on findings with smaller numbers of users [30]. The study will be carried out in five countries, Italy, Northern Ireland, Sweden, Portugal and Lithuania, and so each country will aim to recruit 10 people (i.e. 50 participants in total).

##### Statistical analysis

Statistical analysis will be performed using Microsoft Excel. Appropriate descriptive analyses will be used to summarise participant characteristics and outcomes.

To evaluate feasibility recruitment (% of target number of participants recruited), retention (% of recruited participants with follow-up data) and engagement rates will be reported.

The following criteria would suggest that a main trial is not feasible: observed recruitment rate falls short of 70% of that anticipated, overall dropout of over 35%, no apparent change in the outcomes with confidence intervals that include large negative values, feedback from participants that they were unable to complete (or lack of engagement with) MyRelief and feedback from participants that suggests that the MyRelief content or format was not acceptable or usable.

We will attempt to identify the percentage of people who participate in the intervention by signing up directly to the website versus the percentage who are recruited via the research team. We will use the usability and acceptability data to understand how the intervention was experienced by participants and any changes that they think would improve the intervention.

As this is a feasibility study, significance tests will not be performed on secondary outcomes, such as EQ-5D-3L and ODQ. Intervention effects will be represented by point estimates, and 95% confidence intervals will be estimated at each follow-up time point.

##### Data management and data protection

All data will be collected either by the MyRelief website (clinical endpoints and usability DATASUS, engagement with MyRelief, i.e. units viewed, minutes viewed). All data will be encrypted and saved in a secure online repository held by Lund University, with participant data only accessible by the local research team at each site. All information collected will be kept confidential, all identifiable data will be kept in a locked cabinet and forms with identifiable data will be kept separate from the outcome data. The outcome data will be collected digitally in Excel. Data quality will be enforced by having range checks and valid values. Recordings of qualitative data will be destroyed once transcribed. Any changes to the protocol will be reported to the research ethics committee in each country. The final dataset will be accessed by the principal investigator and the research team. The protocol, the anonymised participant level dataset and any statistical codes used will be made available on request. Comparative uptake and engagement rates will be available via the Udemy MOOC.

## Discussion

This multicenter study will evaluate the feasibility of conducting a randomised controlled trial of a digital intervention for the management of persistent LBP in working adults. If shown to be feasible, one option in terms of delivery will be via a MOOC. The increasing time pressures in the work environment address the extensive need of access to evidence-based knowledge for adults living and working with LBP and require easily accessible solutions. MOOCs can be used targeting a wide audience, but there is limited evidence of their potential to support self-management strategies in LBP. The findings will inform the design of a future randomised controlled trial if it is shown to be feasible.

### Supplementary Information

**Additional file 1. **SPIRIT 2013 Checklist: Recommended items to address in a clinical trial protocol and related documents.

## Data Availability

All materials related to the MyRelief MOOC can be found online at https://www.udemy.com/course/self-management-strategies-for-people-with-low-back-pain/?referralCode=82CB2FEADB8A6BE44B69. For further information of the study, you may contact the PI of the project Eva Ekvall Hansson (eva.ekvall_hansson@med.lu.se).

## Abbreviations

LBP: Low back pain
MOOC: Massive open online course
SPIRIT: Standard Protocol Items: Recommendations for Interventional Trials
